# The role of S*taphylococcus aureus* carriage in the pathogenesis of bloodstream infection

**DOI:** 10.1186/1756-0500-7-428

**Published:** 2014-07-05

**Authors:** Caroline Marshall, Emma McBryde

**Affiliations:** 1 Department of Medicine, University of Melbourne and Victorian Infectious Diseases Service, Royal Melbourne Hospital, Grattan Street, Parkville, Victoria 3050, Australia

**Keywords:** *Staphylococcus aureus*, Blood stream infection, Colonisation

## Abstract

**Background:**

*Staphylococcus aureus* (SA) colonisation is associated with development of bloodstream infection (BSI), with the majority of colonising and infecting strains identical by pulsed-field gel electrophoresis (PFGE). We examined SA colonisation in patients with SABSI to delineate better the relationship between the two.

**Methods:**

Patients with SABSI were swabbed in the nose, throat, groin, axilla and rectum. Isolates were typed using PFGE. Logistic regression was performed to determine factors associated with positive swabs.

**Results:**

79 patients with SABSI had swabs taken. 46 (58%) had ≥ 1 screening swab positive for *S. aureus*; of these 37 (80%) were in the nose, 11 (24%) in the throat, 12 (26%) in the groin, 11 (24%) in the axilla and 8 (17%) in the rectum. On multivariate analysis, days from blood culture to screening swabs (OR 0.5, 95% CI 0.32-0.78, P = 0.003) and methicillin resistance (OR 9.5, 95% CI 1.07-84.73, P = 0.04) were associated with having positive swabs. Of 46 participants who had a blood sample and 1 other sample subtyped, 33 (72%, 95% CI 57-84%) had all identical subtypes, 1 (2%) had subtypes varying by 1–3 bands and 12 (26%) had subtypes ≥ 3 bands different. 30/36 (83%) blood-nose pairs were identical.

**Conclusion:**

Overall, 58% of patients with SABSI had positive screening swabs. Of these, only 80% had a positive nose swab ie less than half (37/79, 47%) of all SABSI patients were nasally colonised. This may explain why nasal mupirocin alone has not been effective in preventing SA infection. Measures to eradicate non-nasal carriage should also be included.

## Background

Colonisation with *Staphylococcus aureus* has a well-recognised association with development of infection, including surgical site and blood stream infections [1,2]. Evidence to confirm this as a causal link has been provided by several studies showing that the majority of colonising and infecting strains are identical when typed using pulsed-field gel electrophoresis (PFGE) [3,4]. However, the detail of these studies indicates that the proportion of participants with no bacteraemia and no colonisation is either high [4] or not given [3]. It is known that individuals can be colonised with *S. aureus* in sites other than the nose, including the throat, axilla, groin and rectum and it is thought that these non-nasal sites might be important in the pathogenesis of infection [5] and also the inconsistent success of intra-nasal mupirocin in preventing infections [1,6]. Gastro-intestinal carriage in particular has been suggested as an important reservoir for *S. aureus* carriage [7,8].

In this study, we swabbed patients with *S. aureus* blood stream infection (SABSI) in multiple sites to determine what proportion was colonised at any site, which sites were most often colonised and whether gastrointestinal carriage in particular was important and whether the colonising and infecting isolates were the same.

## Methods

The study took place at Melbourne Health between 14/05/08 and 11/09/10. The majority of patients were recruited from the Royal Melbourne Hospital (RMH), with a small number from another affiliated hospital. RMH is an adult tertiary referral, university hospital with 350 beds and a 28 bed intensive care unit.

The number of patients needed for this study was calculated by calculating confidence intervals around the possible proportions of patients with *S. aureus* carriage at a particular site. We chose a sample size of 100 bacteraemic patients to ensure the standard deviation of the estimated proportions was 5% or less.

Patients with blood cultures positive for SA were notified to the researcher by the microbiology laboratory. Many were unable to participate in the study because of inability to give consent resulting from illness severity, cognitive impairment or lack of English proficiency with no interpreter available. Patients with neutropenia were excluded because they were unable to have rectal swabs taken because of the potential risk of bacterial translocation [9]. Study participants were swabbed in the nose, throat, groin, axilla and rectum with dry swabs as soon as possible after preliminary blood culture results were available. Data were collected on patient demographics, length of hospital stay prior to blood cultures and screening swabs, intensive care unit admission and intubation status, use of enteric feeding tube, antibiotic and antacid use and use of enteric or topical antibiotics at the time of blood cultures. The likely source of the blood stream infection was determined from the patient notes and if the patient had MRSA, determination of likely community or healthcare acquisition was made. Decolonisation with mupirocin or chlorhexidine body washes was not used on any patients in the hospital at that time.

Swabs were plated onto NC (nalidixic acid and colistin sulphate) plates up to June 2008. SAID chromogenic agar (Biomerieux) plates were then used until the end of September 2009, after which CNA (Colombia horse blood, naladixic acid and Colimycin) plates (Oxoid) were used. Presumptive staphylococcal isolates were tested using latex agglutination and sensitivities were determined on the Vitek 2 analyser. Identification was performed using a DNAse plate, an ORSA plate, an Oxacillin, Esculin plate and a 6.5% salt plate.

Isolates were typed by PFGE using a modified method previously reported [10]. The blood isolate was designated the index subtype and those 1–3 bands different were designated 1a, 1b etc. Those greater than three bands different were designated as a different subtype ie type 2, 3 etc. [11]. Not all isolates were available for subtyping, but denominators are given in the results section.

Data were analysed using Stata version 9 (Stata Corp., College Station, TX, US). Descriptive analysis was performed and 95% confidence intervals calculated around proportions. Univariate logistic regression was performed to determine associations between predictor variables and the outcome variable (positive swabs versus no positive swabs). Stepwise multivariate regression was performed using a P value of 0.1 for entry and a P value of 0.2 for removal from the model.

This study was approved by the Melbourne Health Human Research Ethics Committee and written consent was obtained from the participant or responsible person if the participant was unable to provide consent.

## Results

**Figure 1:**
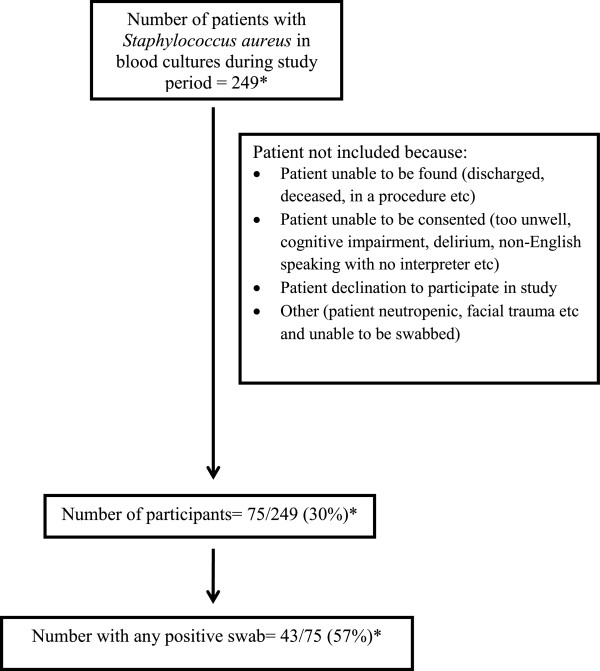
**Flow diagram of patients in study.** *Patients only included once and patients from other institution excluded.

There were 78 patients with *S. aureus* BSI entered in the study, one twice. This was counted as separate entries into the study as the episodes of blood stream infection were over three months apart, making a total of 79 study entries. Patient details were unavailable for the three patients from the affiliated hospital (Figure 1). All participants had swabs taken at all sites. The average age of participants was 60 (median 61, range 19–88) years. Sixty-four (81%) participants were male and 16 (20%) were in the intensive care unit.

Table 1 shows the susceptibility of blood isolates and the timing of isolation. Of the MRSA isolates, four were sensitive to erythromycin, suggesting a community acquired strain although one had its onset ten days after hospital admission. Three of the isolates that were methicillin resistant and erythromycin sensitive, however, had risk factors for healthcare association (a history of either MRSA colonisation or infection in the previous year, hospitalisation, surgery, dialysis or residence in a long-term care facility within the previous year or an indwelling catheter or percutaneous device at the time of the positive blood culture).

**Table 1:** Susceptibility of isolates and timing of isolation

**Isolates**	**Number of isolates (%) (n = 79)**	**Number isolated before or within 2 days of hospital admission (n = 53)**
Penicillin sensitive *S. aureus* (PSSA)	12 (15%)	10 (83%)
Methicillin sensitive *S. aureus* (MSSA)	55 (70%)	38 (70%)
Methicillin resistant *S. aureus* (MRSA)	12 (15%)	5 (45%)

The likely primary source of the BSI is shown in Table 2.

**Table 2:** Likely source of blood stream isolate

**Source**	**Number (n = 79)**	**Percentage**
AV fistula/graft for dialysis	10	12.99
Bone or joint infection	5	6.49
Endocarditis	11	14.29
Intravascular line	30	38.96
Pneumonia	1	1.30
Skin or soft tissue	5	6.49
Surgical site infection	6	7.79
No source identified	7	9.09
Other	2	2.60

AV - arteriovenous.

The median number of days from the first positive blood culture to the date of screening swabs was three (range 1–8). Sixty-four (81%) of participants had screening swabs performed within four days of their first positive blood culture. Forty-six participants (58%, 95% CI 47–69) had at least one screening swab positive for *S. aureus*. Table 3 shows the proportion of screening sites positive for *S. aureus.* The most common combinations of sites were nose-throat, nose-axilla and nose-groin with three each and all sites and throat-groin being positive in two participants each.

**Table 3:** **Screening sites positive for *S. aureus***

**Site**	**Number of participants positive in this site as a proportion of all patients with BSI (%) (n = 78)**	**Number of participants with positive SA swabs in this site as a proportion of all SA swab positive patients (%) (n = 46)**	**95% confidence interval**	**Number of participants with SA positive swabs in this site only (%) (n = 46)**
Nose	37 (47%)	37 (80%)	69-92	21 (46%)
Throat	11 (14%)	11 (24%)	11-37	3 (4%)
Groin	12 (15%)	12 (26%)	12-39	1 (1%)
Axilla	11 (14%)	11 (24%)	11-37	1 (1%)
Rectum	8 (10%)	8 (17%)	6-29	0

Numbers add up to greater than 100% as patients could be positive in more than one site.

BSI – blood stream infection, SA – *Staphylococcus aureus.*

Forty-two (95.5%) patients with positive screening swabs were on antibiotics at the time of screening and all 33 patients with negative screening swabs were on antibiotics at the time of the screening swabs (P = 0.22).

Logistic regression was performed to determine any associations between the outcome variable (any positive swabs versus no positive swabs) and the predictor variables (age, gender, type of plate used [either NC or CNA vs. chromogenic agar], days from blood cultures to screening swabs, methicillin resistance [vs. methicillin sensitivity] and any antibiotics at the time of the screening swabs). On univariate analysis, age (OR 1.03, 95% CI 1.0-1.06, P = 0.048), days from blood cultures to screening swabs (OR 0.52, 95% CI 0.36-0.79, P = 0.002) and methicillin resistance (OR 10.1, 95% CI 1.23-82.33, P = 0.03) were found to be associated with having any positive swabs. On multivariate regression, age was no longer significant (OR 1.03, 95% CI 1.00-1.06, P = 0.6) but days from blood culture to screening swabs remained significant (OR 0.5, 95% CI 0.32-0.78, P = 0.003) as did methicillin resistance (OR 9.5, 95% CI 1.06-84.73, P = 0.04).

Forty-six participants had a *S. aureus* isolate from blood and one or more screening sites subtyped using PFGE. Twenty (44%) had two different samples, 18 (39%) had three, four (9%) had four, two (4%) had five and two (4%) had six samples submitted. Thirty-three (72%) had all identical subtypes, one (2%) had subtypes varying by one to three bands and 12 (26%) had samples with subtypes deviating greater than three bands. In 11 of the 12 patients with different subtypes, only one of the samples differed from all the others. In the one exception, the participant had a blood and groin isolate that were identical, axilla and rectal isolates that were identical to each other but more than three bands different from the blood and groin isolates and a throat isolate that was more than three bands different from each of the other two subtypes. Of the 36 participants that had at least a blood and nose isolate typed, six (17%, 95% CI 6-33%) were greater than three bands different. Of the seven patients that had blood and rectal isolates subtyped, two differed by more than three bands.

## Discussion

This study found that only 58% of patients with a SABSI had a screening swab that was positive at any site (nose, throat, groin, axilla, rectum) and of these, 80% had a positive nose swab. Overall, this means that less than half (37/79, 47%) of all patients with SABSI in this study were found to be colonised in the nose. Of the 36 blood-nose pairs that were subtyped, 30 (83%) had identical nose and bloodstream isolates by PFGE. Patients with MRSA were more likely to have positive screening swabs, suggesting that there may be an intrinsic difference in the propensity for MRSA to colonise body sites compared with MSSA or that the patients with MRSA may have been sicker or hospitalised longer and thus were more likely to develop multisite colonisation. Another explanation may be that once colonised, MRSA are more likely to invade.

In several studies, infecting and colonising isolates have been typed and most isolates have been found to be identical. In one study, 40/3420 prospectively followed nasal *S. aureus* carriers developed nosocomial SABSI compared with 41/10558 non-nasal carriers [4]. Typing using PFGE showed that 32/40 (80%) blood isolates were identical to the nasal strain. However, it is worth noting that 41/81 (51%) of participants with SABSI were not nasally colonised and 49/80 (61%) bacteraemic patients were not nasally colonised with the same *S. aureus* strain, which are almost identical to the figures in our study. That study did not swab non-nasal sites, unlike our study where it was found that 9/42 (20%) of the participants not colonised in the nose were colonised at other sites - these would have been classified as “non-nasal carriers” in that study. These “non-carriers” were found to have a higher mortality from SABSI than the “nasal carriers”; one could speculate whether patients with non-nasal carriage are different from nasal carriers, making it especially important to devise effective decolonisation strategies for non-nasal sites, such as chlorhexidine body washes.

In another study, nasal and blood isolates were typed using PFGE and were found to be identical in 180/219 (82.2%, 95% CI 76.4-87.1) [3]. If areas other than the nose were included, 94.3% were identical. However, the total number of bacteraemic patients who were screened for nasal carriage and the proportion of bacteraemic patients who were nasal carriers of *S. aureus* were not reported. In this study, although nasal swabs were reported to be taken “immediately after the isolation of *S. aureus* from the blood”, no data were collected on the actual time to swab taking and whether the patient was already on antibiotics at the time of swab collection.

Non-nasal colonisation is well recognised for *S. aureus*, including MRSA, in many cases without concurrent nasal colonisation [12]. These non-nasal sites have been postulated to be important in the pathogenesis of infection [7,8], as a reservoir for transmission [13] and as a potential explanation for failure of nasal decolonisation in preventing infection with *S. aureus*[14]. The throat has been found to be an important site of colonisation, particularly for hospitalised patients with MRSA [15]. The gastrointestinal tract has also been recognised as a reservoir for *S. aureus*[16,7-22], with studies showing increased sensitivity for detection of MRSA when rectal swabs were included [23], rectal colonization without nasal carriage [8,18,24] and increased chance of developing *S. aureus* infection in rectal carriers [7,8].

We have previously found that 6.3% and 37.5% of methicillin-sensitive (MSSA) and methicillin-resistant *S. aureus* (MRSA) carriers in an ICU population were colonised in the rectum [25]. In this study, swabbing the nose, throat and rectum and the nose, throat and axilla were the most sensitive combination of sites for detecting MRSA and MSSA respectively. Although groin/perineal swabs have been assumed to be synonymous with rectal carriage [26], we found that 3/8 rectal carriers were negative in groin swabs and 7/12 with positive groin swabs had negative rectal swabs, suggesting that these cannot be used interchangeably. In the current study, we found that 17% of participants were colonised in the rectum.

Because of the strong association of nasal carriage of *S. aureus* with development of infection, it could be reasonably assumed that eradication of nasal carriage would reduce the incidence of infection. However, several major randomised double-blind placebo-controlled trials have not shown that intranasal mupirocin prevented surgical site infection or non-surgical site infection [1,6,27]. A randomised placebo-controlled trial using intranasal mupirocin and chlorhexidine body washes for eradication of endemic MRSA colonisation at multiple body sites in ICU patients showed there was no significant difference in eradication of MRSA at any site or in the rate of MRSA infection. Risk factors for failure to eradicate MRSA included multi-site MRSA carriage [28].

Another more recent study showed that use of nasal mupirocin and daily chlorhexidine body washes prevented surgical and non-surgical site infections in *S. aureus* carriers identified using rapid detection methods [29]. In other uncontrolled studies, mupirocin has been used successfully to eradicate carriage of *S. aureus* but recolonisation has been a problem [5,12,24]. Other hidden reservoirs, such as the throat and gastrointestinal tract, could contribute to the failure of mupirocin to completely prevent infection [5]. Enteral vancomycin has been used in several uncontrolled trials with consequent reductions in MRSA carriage and infection in the outbreak and the endemic setting [19,30]. Although concerns about the development of resistance prevent this from becoming a widespread intervention, it suggests that gastrointestinal carriage may be important.

Although this study included a heterogeneous mixture of patients with MSSA and MRSA and both community and healthcare associated infections, we have found results consistent with other studies. It would have been ideal to swab patients and follow them up prospectively to determine development of BSI, but the large numbers (several thousand) required to conduct such a cohort study [4] was not feasible. Other studies have based their findings on similar methodology of screening patients after results were available [3]. We tried to swab patients as soon as possible after results were available.

Antibiotic treatment may have decreased the yield of detecting colonisation, although in the multivariable analysis, this was not a significant predictor of whether screening swabs were positive or not. However, carriage was still detected in many patients on antibiotics suggesting only variable efficacy in eliminating carriage using antibiotics, and this is consistent with other studies where *S. aureus* was still able to be cultured from screening swabs from many patients on antibiotics [31].

Some studies have found using flocked swabs and pre-moistened swabs increases the yield [32] but this has not been found universally [33,34]. We also did not use broth enrichment when processing screening swabs which also may have decrease the yield. Interestingly, the published studies examining colonisation and bacteraemia did not report how screening swabs were processed or whether patients were on antibiotics at the time of taking swabs [3,4].

## Conclusion

In conclusion, this study confirms that patients with SABSI are colonised in multiple sites, including the gastrointestinal tract. Consistent with other studies, we also found that the majority of patients with blood stream infection were not colonised with the same strain in the nose, possibly explaining why nasal mupirocin alone has not been shown to be effective in the prevention of infection. Measures to eradicate carriage at these non-nasal sites in addition to nasal sites, such as chlorhexidine body washes, may be considered to prevent infection, especially for patients with MRSA. Further research into decolonisation of non-nasal and non-skin sites, especially the gastrointestinal tract, is also needed, as eradication at these sites may provide even more effective infection prevention strategies.

## Competing interests

The authors declare that they have no competing interests.

## Authors’ contributions

CM conceived the study, acquired and analysed the data and wrote the report. EM assisted with study design, data analysis and review of the manuscript. Both authors read and approved the final manuscript.

## Authors’ information

CM is an infectious diseases physician at the Victorian Infectious Diseases Service at the Royal Melbourne Hospital and Principal Research Fellow at the Department of Medicine, University of Melbourne. She has an interest in infection control and hospital epidemiology and antimicrobial stewardship. EM is an infectious diseases physician and Head of Epidemiology at the Victorian Infectious Diseases Service at the Royal Melbourne Hospital. She had an interest in mathematical modelling.

## References

[B1] Perl TM Cullen JJ Wenzel RP Zimmerman MB Pfaller MA Sheppard D Twombley J French PP Herwaldt LA Intranasal mupirocin to prevent postoperative *Staphylococcus aureus* infections N Engl J Med 2002 346 1871 1877 10.1056/NEJMoa003069 12063371

[B2] Pujol M Peña C Pallares R Ariza J Ayats J Dominguez MA Gudiol F Nosocomial *Staphylococcus aureus* bacteremia among nasal carriers of methicillin-resistant and methicillin-susceptible strains Am J Med 1996 100 509 516 10.1016/S0002-9343(96)00014-9 8644762

[B3] Von Eiff C Becker K Machka K Stammer H Peters G Nasal carriage as a source of *Staphylococcus aureus* bacteremia N Engl J Med 2001 344 11 16 10.1056/NEJM200101043440102 11136954

[B4] Wertheim HFL Vos MC Ott A Van Belkum A Voss A Kluytmans JAJ Van Keulen PHJ Vandenbroucke-Grauls CMJE Meester MHM Verbrugh HA Risk and outcome of nosocomial *Staphylococcus aureus* bacteraemia in nasal carriers versus non-carriers Lancet 2004 364 703 705 10.1016/S0140-6736(04)16897-9 15325835

[B5] Peña C Fernández-Sabe N Domínguez MA Pujol M Martinez-Castelao A Ayats J Gudiol F Ariza J *Staphylococcus aureus* nasal carriage in patients on haemodialysis: role of cutaneous colonization J Hosp Infect 2004 58 20 27 10.1016/j.jhin.2004.04.018 15350709

[B6] Wertheim HFL Vos MC Ott A Voss A Kluytmans JAJW Vandenbroucke-Grauls CMJE Meester MHM Van Keulen PHJ Verbrugh HA Mupirocin prophylaxis against nosocomial *Staphylococcus aureus* infections in nonsurgical patients Ann Int Med 2004 140 419 425 10.7326/0003-4819-140-6-200403160-00007 15023707

[B7] Ray AJ Pultz NJ Bhalla A Aron DC Donskey CJ Coexistence of vancomycin-resistant enterococci and *Staphylococcus aureus* in the intestinal tracts of hospitalized patients Clin Infect Dis 2003 37 875 881 10.1086/377451 13130397

[B8] Squier C Rihs JD Risa KJ Sagnimeni A Wagener MM Stout J Muder RR Singh N *Staphylococcus aureus* rectal carriage and its association with infections in patients in a surgical intensive care unit and a liver transplant unit Infect Control Hosp Epidemiol 2002 23 495 501 10.1086/502095 12269445

[B9] Meckler G Lindemulder S Fever and neutropenia in pediatric patients with cancer Emerg Med Clin North Am 2009 27 525 544 10.1016/j.emc.2009.04.007 19646652

[B10] Struelens MJ Deplano A Godard C Maes N Serruys E Epidemiologic typing and delineation of genetic relatedness of methicillin-resistant *Staphylococcus aureus* by macrorestriction analysis of genomic DNA using pulsed-field gel electrophoresis J Clin Micro 1992 30 2599 2605 10.1128/jcm.30.10.2599-2605.1992 PMC270485 1328279

[B11] Tenover FC Arbeit RD Goering RV Mickelsen PA Murray BE Persing DH Swaminathan B Interpreting chromosomal DNA restriction patterns produced by pulsed-field gel electrophoresis: Criteria for bacterial strain typing J Clin Microbiol 1995 33 2233 2239 7494007 10.1128/jcm.33.9.2233-2239.1995 PMC228385

[B12] Paterson DL Rihs JD Squier C Gayowski T Marino IR Sagnemeni A Singh N Lack of efficacy of mupirocin in the prevention of infections with methicillin-resistant *Staphylococcus aureus* in liver transplant recipients and candidates Transplantation 2003 75 194 198 10.1097/01.TP.0000040602.01701.85 12548122

[B13] Boyce JM Havill NL Maria B Frequency and possible infection control implications of gastrointestinal colonization with methicillin-resistant *Staphylococcus aureus* J Clin Micro 2005 43 5992 5995 10.1128/JCM.43.12.5992-5995.2005 PMC1317179 16333087

[B14] Wertheim HFL Verveer J Boelens HAM Van Belkum A Verbrugh HA Vos MC Effect of mupirocin treatment on nasal, pharyngeal, and perineal carriage of Staphylococcus aureus in healthy adults Antimicrob Agents Chemother 2005 49 1465 1467 10.1128/AAC.49.4.1465-1467.2005 15793127 PMC1068605

[B15] Marshall C Spelman D Is throat screening necessary to detect methicillin-resistant staphylococcus aureus colonization in patients upon admission to an intensive care unit? J Clin Microbiol 2007 45 3855 10.1128/JCM.01176-07 17728478 PMC2168532

[B16] Brodie J Kerr MR Sommerville T The hospital staphylococcus. A comparison of nasal and faecal carrier states Lancet 1956 1 19 20 13279153 10.1016/s0140-6736(56)91855-4

[B17] Matthias JQ Shooter RA Williams REO Staphylococcus aureus in the faeces of hospital patients Lancet 1957 1 1172 1173 13439996 10.1016/s0140-6736(57)91740-3

[B18] Rimland D Roberson B Gastrointestinal carriage of methicillin-resistant *Staphylococcus aureus* J Clin Microbiol 1986 24 137 138 3722359 10.1128/jcm.24.1.137-138.1986 PMC268848

[B19] Silvestri L Milanese M Oblach L Fontana F Gregori D Guerra R Van Saene JKF Enteral vancomycin to control methicillin-resistant *Staphylococcus aureus* outbreak in mechanically ventilated patients Am J Infect Control 2002 30 391 399 10.1067/mic.2002.122255 12410215

[B20] Crossley K Solliday J Comparison of rectal swabs and stool cultures for the detection of gastrointestinal carriage of *Staphylococcus aureus* J Clin Micro 1980 11 433 434 10.1128/jcm.11.4.433-434.1980 PMC273420 6989861

[B21] Trick WE Weinstein RA DeMarais PL Kuehnert MJ Tomaska W Nathan C Rice TW McAllister SK Carson LA Jarvis WR Colonization of skilled-care facility residents with antimicrobial-resistant pathogens J Am Geriatr Soc 2001 49 270 276 10.1046/j.1532-5415.2001.4930270.x 11300237

[B22] Boyce JM Havill NL Kohan C Gumigan DG Ligi CE Do infection control measures work for methicillin-resistant *Staphylococcus aureus*? Infect Control Hosp Epidemiol 2004 25 395 401 10.1086/502412 15188845

[B23] Batra R Eziefula AC Wyncoll D Edgeworth J Throat and rectal swabs may have an important role in MRSA screening of critically ill patients Intensive Care Med 2008 34 1703 1706 10.1007/s00134-008-1153-1 18500421

[B24] Dupeyron C Campillo B Bordes M Faubert E Richardet J-P Mangeney N A clinical trial of mupirocin in the eradication of methicillin-resistant *Staphylococcus aureus* in a digestive disease unit J Hosp Infect 2002 52 281 287 10.1053/jhin.2002.1287 12473473

[B25] Verhallen A Kooistra M van Jaarsveld B Cannulating in haemodialysis: rope-ladder or buttonhole technique? Nephrol Dial Transplant 2007 22 2601 2604 10.1093/ndt/gfm043 17557776

[B26] Acton DS Tempelmans Plat-Sinnige MJ van Wamel W de Groot N van Belkum A Intestinal carriage of *Staphylococcus aureus*: how does its frequency compare with that of nasal carriage and what is its clinical impact? Eur J Clin Microbiol Infect Dis 2009 28 115 127 10.1007/s10096-008-0602-7 18688664

[B27] Kalmeijer MD Coertjens H Van Nieuwland-Bollen PM Bogaers-Hofman D De Baere GAJ Stuurman A Van Belkum A Kluytmans JAJ Surgical site infections in orthopedic surgery: the effect of mupirocin nasal ointment in a double-blind, randomized, placebo-controlled study Clin Infect Dis 2002 35 353 358 10.1086/341025 12145715

[B28] Harbarth S Dharan S Liassine N Herrault P Auckenthaler R Pittet D Randomized, placebo-controlled, double-blind trial to evaluate the efficacy of mupirocin for eradicating carriage of methicillin-resistant *Staphylococcus aureus* Antimicrob Agents Chemother 1999 43 1412 1416 10348762 10.1128/aac.43.6.1412 PMC89288

[B29] Bode LG Kluytmans JA Wertheim HF Bogaers D Vandenbroucke-Grauls CM Roosendaal R Troelstra A Box AT Voss A van der Tweel I van Belkum A Verbrugh HA Vos MC Preventing surgical-site infections in nasal carriers of Staphylococcus aureus N Engl J Med 2010 362 9 17 10.1056/NEJMoa0808939 20054045

[B30] de la Cal MA Cerdá E Van Saene JKF García-Hierro P Negro E Parra ML Arias S Ballesteros D Effectiveness and safety of enteral vancomycin to control endemnicity of methicillin-resistant *Staphylococcus aureus* in a medical/surgical intensive care unit J Hosp Infect 2004 56 175 183 10.1016/j.jhin.2003.09.021 15003664

[B31] Shenoy ES Noubary F Kim J Rosenberg ES Cotter JA Lee H Walensky RP Hooper DC Concordance of PCR and culture from nasal swabs for detection of methicillin-resistant staphylococcus aureus in a setting of concurrent antistaphylococcal antibiotics J Clin Microbiol 2014 52 1235 1237 10.1128/JCM.02972-13 24452168 PMC3993487

[B32] Verhoeven P Grattard F Carricajo A Pozzetto B Berthelot P Better detection of Staphylococcus aureus nasal carriage by use of nylon flocked swabs J Clin Microbiol 2010 48 4242 4244 10.1128/JCM.01425-10 20844232 PMC3020835

[B33] De Silva S Wood G Quek T Parrott C Bennett CM Comparison of flocked and rayon swabs for detection of nasal carriage of Staphylococcus aureus among pathology staff members J Clin Microbiol 2010 48 2963 2964 10.1128/JCM.01617-09 20504992 PMC2916558

[B34] Codrington L Kuncio D Han J Nachamkin I Tolomeo P Hu B Lautenbach E Yield of methicillin-resistant Staphylococcus aureus on moist swabs versus dry swabs Am J Infect Control 2013 41 469 470 10.1016/j.ajic.2012.09.011 23337302

